# Designing Opioids That Deter Abuse

**DOI:** 10.1155/2012/282981

**Published:** 2012-11-08

**Authors:** Robert B. Raffa, Joseph V. Pergolizzi, Edmundo Muñiz, Robert Taylor, Jason Pergolizzi

**Affiliations:** ^1^Department of Pharmaceutical Sciences, School of Pharmacy, Temple University, Philadelphia, PA 19140, USA; ^2^Department of Medicine, School of Medicine, Johns Hopkins University, Baltimore, MD 21205, USA; ^3^Department of Anesthesiology, School of Medicine, Georgetown University, Washington, DC 20057, USA; ^4^Kirax Corporation, Inc., Bonita Springs, FL 34134, USA; ^5^NEMA Research Inc., 840 111th Avenue North, Naples, FL 34108, USA

## Abstract

Prescription opioid formulations designed to resist or deter abuse are an important step in reducing opioid abuse. In creating these new formulations, the paradigm of drug development target should be introduced. Biological targets relating to the nature of addiction may pose insurmountable hurdles based on our current knowledge and technology, but products that use behavioral targets seem logical and feasible. The population of opioid abusers is large and diverse so behavioral targets are more challenging than they appear at first glance. Furthermore, we need to find ways to correlate behavioral observations of drug liking to actual use and abuse patterns. This may involve revisiting some pharmacodynamic concepts in light of drug effect rather than peak concentration. In this paper we present several new opioid analgesic agents designed to resist or deter abuse using physical barriers, the inclusion of an opioid agonist or antagonist, an aversive agent, and a prodrug formulation. Further, this paper also provides insight into the challenges facing drug discovery in this field. Designing and screening for opioids intended to resist or deter abuse is an important step to meet the public health challenge of burgeoning prescription opioid abuse.

## 1. Introduction

A fundamental tenet and driving force of drug discovery is that there is a clear and important *medical need* for which we can identify a *biological target*. Success in drug discovery is measured insofar as this medical need is adequately addressed to the extent that our current understanding of basic science and existing technology permits. The biological target can take many forms. For example, in the case of a new angiotensin-converting enzyme (ACE) inhibitor for hypertension, there is both a clear medical need (essential hypertension) and an obvious biological target (inhibitor of the enzyme). A more complex example might be a new drug for the amelioration of symptoms of Alzheimer's disease. The medical need for such a drug is clear, but the biological target may be only hypothetical. The justification for initiating drug discovery in this case is clear, even if the outcome is less so. Another example might be a novel insulin delivery system that provides insulin release in a manner that more closely matches blood glucose levels. In this example, the medical need is valid and the discovery target merges biological with technological principles. When it comes to designing and screening for opioids that deter abuse, the same principles should be applied, but formulators should be aware of medical needs unlike the previous examples. What are the medical needs, and, if so, what is the target?

At first, the answers might appear obvious. Prescription opioid analgesics are abused, and that abuse has negative medical and even societal consequences. It might be possible to discover opioid drugs that have less abuse liability or that are designed in a formulation that is more resistant to abuse. The first of these targets—an opioid with a *lower abuse liability*—currently seems less immediate. Depending on receptor and 2nd-messenger transduction processes, this approach is the more difficult path, but it is possible. For example, opioids with an agonist/antagonistic mechanism such as nalbuphine and buprenorphine were originally developed with the goal of reducing abuse liability. Discovery of additional agonist/antagonist opioids is very possible. The second target, namely, to design an opioid formulation that can *deter abuse*, seems quite attainable with the creative application of new technologies but still may not be enough to prevent abusers who are determined to circumvent the new barriers. Ideally, the perfect opioid would be one that delivers optimal therapeutic benefit and optimal abuse deterrence, and thus formulators should work with both goals in mind in order to satisfy both medical needs. The history of the medical use of opioids has been an interesting exercise in attempting to balance the benefits of these drugs with their associated risks.  Table 1 describes events that have spurred opioid abuse as well as the events to prevent such abuse.

In this paper, we review these new technologies, but, perhaps just as importantly, we ask the question whether there is an actual medical need for these products. To answer this question, we will examine the postulated target population(s), the likely success in addressing what may be separate problems of prescription versus illicit abuse, and some of the solutions to the abuse dilemma. Thus, we attempt to apply the same criteria that would be used with a more conventional drug discovery decision.

## 2. Opioid Abuse

Opioid abuse can have both negative medical and societal impacts. One of the biggest concerns to date is the growing number of deaths associated with opioid overdose. From 1999 to 2008, the United States of America has seen a substantial increase in overdose related deaths [1]. The death rate due to overdose in 2008 was four times as much as in 1999, and in some states overdose-related deaths are currently outpacing the number of deaths related to motor vehicle accidents. In addition, morbidity has increased as well. Emergency department visits related to the nonmedical use of opioids has doubled between 2004 and 2008 [1]. In turn, the fear of promoting abuse by prescribing patients opioids may cause some physicians to deny a patient of needed pain therapy.

## 3. Target Populations

The Controlled Substances Act requires that patterns of drug abuse be evaluated when considering a drug's abuse potential [2]. With more than 35 million Americans having used prescription opioids nonmedically [3], these patterns are diverse. Even the terminology describing these behaviors is controversial. We use the term *abuse* rather than misuse, inappropriate use, or nonmedical use, because the Diagnostic and Statistical Manual of Mental Disorders or DSM-IV uses “substance abuse” to describe “a maladaptive pattern of substance use manifested by recurrent and significant consequences related to the repeated use of substances” [4], and the Food and Drug Administration (FDA) uses the term “abuse” with respect to the products discussed in our paper. It should be noted that definitions related to substance-use disorders are underway for DSM-V, and definitions presented in this paper may not reflect future literature.

There exist several clinically distinguishable categories of prescription opioid abuser, including those with and without legitimate prescriptions [5]. Each subpopulation has its own motivations for taking opioids, preferred drugs and routes of administration, and specific behaviors. However, our knowledge about these populations has gaps. For example, while some sources say that most people who abuse prescription opioids obtain them from friends or relatives [3], the National Addiction Vigilance Intervention and Prevention Program (NAVIPPRO) monitoring system found that just as many abusers obtain their drugs from dealers [6]. Purchasing patterns vary by drug; hydrocodone and oxycodone are available about equally from friends and dealers, but morphine, methadone, and fentanyl are mostly obtained from dealers [6]. We have identified the following subpopulations of opioid abusers.

### 3.1. Opportunistic or Recreational Abuser

These individuals take prescription opioids for recreation, to pursue a high, or for experimentation. They may have limited opioid experience, often combine drugs, and rarely inject them [7].

### 3.2. Chronic Pain Patient

Diagnosed chronic pain patients make up less than 1% of the insured population in the United States but consume about 45% of all prescription opioids [8]. It has been estimated that up to 40% of pain patients on chronic opioid therapy display aberrant drug-related behaviors (Table 2) [9], but it is unclear to what extent aberrant drug-taking behaviors predict abuse although some experts believe they do [9, 10]. Chronic pain has been intertwined with substance abuse: 33% of individuals in a substance abuse program reported suffering from chronic pain and individuals in substance abuse treatment programs with chronic pain were significantly more likely to abuse opioids than those not reporting chronic pain (20% versus 8%, *P* < 0.001) [6]. The term *rational abuse* has been put forth to describe chronic pain patients who abuse opioids because of undertreated pain [11], but very little is known about this population.

Chronic opioid users will almost always develop physical dependence while those with drug abuse histories and other predisposing genetic or mental conditions may go on to become addicts. However, it should be made clear that physical dependence is not necessary for addiction. Physical dependence and addiction have two different definitions even though sometimes in the literature they have been used interchangeable. Physical dependence is usually defined as a physical state of adaption to a drug or substance while a person is said to be an addict when the use of the drug leads to personal harm or severe consequences. Chronic pain patients using opioids for long periods of time may first experience physical dependence which may later develop into addiction if patients have other underlying genetic predispositions, psychological conditions, or abuse history.

### 3.3. Persistent Drug Abuser

Persistent or habitual opioid abusers, the best studied of the subpopulations, are those whose opioid abuse is part of their lifestyle. They exhibit some unique behaviors. A study of 9 healthy prescription opioid abusers compared in a double-blind study to 9 nonopioid abusers found that abusers self-administered oxycodone during experimentally induced pain and at other times, while nonabusers only self-administered oxycodone during experimentally induced pain although the subjective effects of oxycodone were similar in both groups [12]. This suggests that subjective effects may not correlate with subsequent behaviors. Abusers were more likely to report that oxycodone made them feel sociable and talkative while nonabusers given opioids were more likely to say the drug made them feel less alert [12]. Unpleasant side effects are more likely to be reported by nonabusers than abusers [13, 14].

#### 3.3.1. Opiate Abuser

An opioid abuser (such as a heroin addict) changes drugs (prescription opioid) if supply is compromised. These abusers select opioids with a rapid onset of action and intense effect [7].

#### 3.3.2. Polydrug Abuser

Polydrug abuser deliberately takes multiple drugs, frequently combines them, and has no clear preferences [7].

#### 3.3.3. Genetic Abuser

An abuser who can become addicted to any type of drug or substance due to having the disease of addiction (Table 2). Abuse-deterrent formulations may not work well in this group and may just push these abusers to a different drug or substance [15]. Treatment in this group should focus on identifying the root causes of addiction.

### 3.4. Rave Abusers

The recently defined “rave” abuser is a club scene denizen seeking a long-duration high [7]. Rapid onset of action is inconsequential, and opioid abuse may be sporadic.

### 3.5. Those with Comorbid Mental Health Disorders or Substance Abuse

Comorbidities are common among those who abuse prescription opioids: 85% or more suffer chronic pain, 55% or more have mental disorders [8], about 40% [8] to 56% [16] have concurrent alcohol dependence, and 60% or more are nicotine dependent [17]. In addition, chemical coping has been applied to those who take prescribed or illegally obtained opioids to address an underlying mental health disorder [11]. They are normally considered the middle ground group, caught between frank addiction and regimen adherence [18]. They have a tendency to focus on the pharmacologic treatment of pain and disregard nonpharmacologic options for pain control (e.g., physical therapist or psychiatrist). This tendency causes patients to sometimes use medications in nonprescribed ways including self-medication by escalating the dose themselves or under times of stress use medications to cope with their problems. This group is not well studied in the literature even though they make up approximately 35% of chronic pain patients. This type of patient may not benefit greatly from abuse deterrent formulations but require psychotherapy to address the underlying mental condition or problem causing opioid misuse.

## 4. Opioid Attributes Liked/Disliked by Abusers

To find development targets for abuse-deterrent opioids, we need to understand what attracts abusers to particular opioids. Screening tools to help predict risk of opioid abuse have been developed [46–48] and shed light on some drug-related behaviors. Other insight comes from clinical trials enrolling opioid abusers, who may be less than truthful with investigators. In one study, nearly a quarter of respondents reported that they had used a product (fentanyl matrix transdermal system) that was not available at the time of the study [49]. Dose-effect response studies of healthy drug abusers are often generalized although their predictive value in other populations is unclear [7]. Further study is warranted. In the following sections descriptions of attributes that may contribute to drug liking are presented.

### 4.1. Drug Delivery Systems

In a study of drug preferences among recreational abusers (*n* = 42), oral tablets generally ranked higher than transdermal patches, but some transdermal patches (fentanyl) rated ahead of some tablets [50–52]. In other drug preference studies, matrix-type patches were preferred by 60% of the recreational users over gel patches [51]. Thus, delivery systems may play a part in selecting opioids for misuse.

### 4.2. Agent

Studies to determine the most frequently abused opioids have had mixed results. According to NAVIPPRO, the most frequently abused prescription opioids in their survey (*n* = 41, 923 cases) are hydrocodone and oxycodone, followed by morphine and methadone [6]. Among oxycodone abusers, the use was roughly evenly split between immediate-release and extended-release formulations [6]. On the other hand, most morphine abusers preferred an extended-release formulation [6]. These results were supported by a survey of 1,818 prescription-opioid-dependent patients entering drug treatment programs, in which 75% reported oxycodone or hydrocodone as their preferred drug, with less than 5% naming fentanyl as drug of choice [17]. However, in a study of recreational drug abusers in Canada (*n* = 42), fentanyl was considered a highly desirable drug (oral fentanyl rated higher than the patch but both were considered more desirable than oxycodone tablets) [53]. Fentanyl is also one of the most frequently abused opioids among US healthcare professionals [53–55]. From these reports, it appears that drug preferences in specific subpopulations may emerge due to a variety of factors such as familiarity with the agent, accessibility, price, ability to conceal the drug, and reputation of the agent within that population. For example, hydrocodone and oxycodone in 2009 were the most highly prescribed opioids (84.9% of total opioids prescribed), and preference for these drugs may be due to their ease of access [56].

### 4.3. Product Attributes

In a survey of 491 self-reported recreational opioid users, 113 product attributes were evaluated, of which those that made an agent attractive were ease of extraction, duration of effect, and rapid onset. Withdrawal effects, injection issues (pain, slow onset), formulation deterrents (adulterants, difficulty extracting drug), slow onset of action, and unpleasant administration made an opioid unattractive [57]. When these features were tested among another group of recreational opioid users (*n* = 564) in terms of what drugs they had *actually used*, the model, though imperfect, showed some good correlations. An abuse-deterrent formulation of pentazocine and naloxone (Talwin) had the least-attractive attributes and was also rarely abused by respondents (less than 1% had ever used it). 

### 4.4. Long-Acting versus Short-Acting Opioids

As a rule of thumb, the fastest to slowest delivery methods for onset of action are inhalation, intravenous (IV), intranasal, transmucosal, and oral [5]. Long-acting opioids have been thought to have a lower abuse potential than short-acting opioids, but a randomized, double-blind, and placebo-controlled crossover study of extended-release morphine versus hydrocodone/acetaminophen (*n* = 18) found that long-acting opioids do not have lower abuse potential than either short-acting opioids or placebo [58]. 

## 5. Other Opioid Attributes That May Promote Abuse

The *abuse liability* of a drug is generally considered the degree to which repeated consumption will occur because of its positive subjective effects, reinforcing effects or to avoid negative effects. It differs from *drug liking*, which is a subjective scoring system for positive and negative effects associated with a given drug [59]. Both abuse liability and drug liking are influenced by many factors, including drug formulation (onset of action, duration of action, and intensity of effect), cost, availability, social acceptability [60], and even popularity among peers [17].

Pharmacokinetic attributes of an agent, including drug absorption, bioavailability, lipophilicity, and metabolism, may affect its likability [61]. Opioid attractiveness has been based in part on how rapidly peak plasma concentration (*C*_max⁡_) is reached [5]. It may be more appropriate to think of opioids in terms of their *peak effects* and the *time to maximal effect* rather than *T*_max⁡_ (time to maximum plasma concentration). In a study of IV administration of oxycodone, hydrocone, and morphine (5, 10, and 20 mg/10/mL infused over five minutes) to 11 recreational nondependent opioid users, pharmacodynamic effects of all agents were observed within five minutes of IV administration, and physiological effects were more prolonged than subjective effects [62]. The subjective effects of hydrocodone dissipated more rapidly than those of oxycodone or morphine although the physiological effects were similar [62]. In preclinical studies, the time required to achieve peak plasma concentrations for IV cocaine and IV hydromorphone are similar [63, 64], but cocaine is more rapidly transported to the brain [61]. Thus, peak plasma and peak effect may occur at different times and for studies of opioid abuse, time to maximum effect is the more relevant variable. A study of an extended-release morphine/naltrexone capsule (ALO-01, Alpharma) reported the *maximum effect* or *E*_max⁡_ value of the agent (both intact and crushed) versus morphine sulfate solution 120 mg based on pupillometry. The morphine solution had a significantly greater *E*_max⁡_ than both intact or tampered ALO-01 (*P* < 0.001) [65]. The area under the effect (AUE) should be considered in abuse-deterrent formulations.

## 6. Products Designed to Resist or Deter Abuse

While abuse-deterrent or abuse-resistant labeling requires large-scale epidemiological studies, which have not yet been conducted, the theoretical value of these formulations has already been recognized by clinicians, insurance carriers, and pharmacy managers [66]. The main approaches to the problem thus far have been a physical barrier, an agonist or antagonist that is activated when the product is altered, an aversive agent, and, most rarely, a prodrug formulation. A barrier may be either physical or pharmacological and both may provide a way to prevent consumption through alternate, nonintended routes. A physical barrier can be composed of a high viscosity gel, which can prevent crushing and may be resistant to aqueous extraction. However, active ingredient may be released by mechanical erosion or enzymatic degradation. In addition, the active ingredient may be encapsulated within an insoluble coating. Pharmacological barriers may consist of a sequestered opioid antagonist or an aversive agent.  Table 3 lists opioid analgesics designed to resist or deter abuse that currently are on the market or in development.

These products are new formulations of well-known agents, such as the new formulation of OxyContin, which releases from 21% to 48% less opioid when tampered (milled, manually crushed, dissolved, and boiled) than the original version [29].

One of the first formulations intended to reduce abuse was a buprenorphine/naloxone formulation (Suboxone). In opioid addicts, buprenorphine/naloxone produces no euphoria [67]. However, in opioid-naïve individuals, buprenorphine/naloxone may produce euphoria when injected [68, 69]. This parallels findings of oral oxycodone, hydrocodone, and hydromorphone which produced unpleasant effects only in those with limited opioid experience, not in experienced users [70]. Drugs with sequestered naloxone or naltrexone can precipitate withdrawal and may be tampered even by patients who understand the risks [71].

In drug discovery for abuse-deterrent opioids, creating the least undesirable product by abusers possible should be the goal. This will require drug developers to understand both how and why certain opioids are diverted by specific abuser populations.

## 7. Real-World Impact of Abuse-Deterrent and Abuse-Resistant Formulations

Drug liking and abuse liability are useful concepts, but we do not fully understand the strength of correlation to *drug using*. Even if drug liking is the correct target, we do not know, for example, how it correlates to use. For example, it would be useful to know that if we could decrease drug liking by 20%, we could reduce abuse of that drug by a given percentage.

Behavioral targets for drug development seem practical, but further study is needed, in particular of opioid abusers who are rarely included in current studies (those with a major psychiatric comorbidity, chronic pain patients, and those with another substance abuse problem) and populations of sporadic users who may go unrecognized (the chemical copper, the rave abuser). What is known about opioid abuser behavior should be correlated against actual use patterns when possible.

At the core of this problem is the nature of addiction itself, which has both neurobiological and psychological components and remains to be more clearly elucidated. Biological targets for the development of new abuse-deterrent opioids are beyond the scope of this paper but remain an important goal for future research. Our current approach to opioid pharmacodynamics requires some retooling to address opioid abuse. For example, a better understanding of *maximum effect* and *time to maximum effect* rather than *C*_max⁡_ and *T*_max⁡_ values may be helpful in this context.

Four main models for abuse-deterrent opioids exist currently; those with a physical barrier (which do not prevent the drug from being abused by those who take it intact, but do make it difficult to snort, smoke, or inject the drug); those with an opioid agonist or antagonist that is released when the drug is misused; those with an aversive agent (niacin); and prodrug, a compound that must undergo a chemical change within the body before becoming active. The list of products in Table 3 is complete to the extent of our knowledge. As these products come into more widespread use, actual experience with the drugs will help shape the next generation of products.

Of course, the authors do not believe that abuse-deterrent opioids will end opioid abuse. The goals of abuse-deterrent opioid formulations are limited and specific to the agents studied. It may be that abuse-deterrent formulations will simply lead to new drug choices by abusers. Surprisingly, there appears to be some degree of “brand loyalty” among recreational abusers in that some products are so well liked that online forums (Opiophile.com, Topix.com opioid forum, Prescriptiondrug-info.com, etc.) discuss how to circumvent tamper-proof mechanisms. That actual real-world effect of these drugs remains to be seen.

## 8. Conclusion

Prescription opioid misuse and abuse is a serious and pervasive public health crisis. Creating abuse-deterrent opioid formulations may be an important step in combating opioid abuse. Creating products based on behavior targets seems feasible, if complex, because of the diversity of populations who abuse opioids. Pharmacodynamic concepts are helpful in the creation of such new formulations but may need to be refined to be more specific to abuser populations. For example, drug liking can be measured, but it is unclear how to correlate this directly to actual drug abuse. Time to maximum effect may be a more useful metric for abuse-deterrent products than maximum serum concentration. Several abuse-deterrent opioid formulations are on the market or in development. Real-world experience with these formulations and ongoing efforts to better understand metrics associated with abuse liability (drug craving and drug liking) are needed to help guide and inform further efforts in creating abuse-deterrent opioid products.

## Figures and Tables

**Table 1:** A very short modern history of opioid analgesia [19].

Year	Event
1804	Morphine isolated from opium (Germany)
1827	Morphine commercially available (Merck)
1832	Codeine isolated (France)
1857	Hypodermic needle invented
1890	First USA law regulating narcotics, a tax on opium and morphine. Narcotics can be freely bought and sold
1903	Heroin addiction is recognized as a major public health crisis in USA
1905	USA bans opium
1914	Harrison Narcotics Act requires registration of physicians, pharmacists, and others associated with narcotics prescribing and distribution
1914	Oxymorphone synthesized (Germany)
1916	Oxycodone synthesized (Germany)
1923	First US federal drug agency (US Treasury Department's Narcotics Division) bans sale of all narcotics in USA
1930	Federal Bureau of Narcotics established in the Treasury Department
1939	Oxycodone available in USA
1959	Oxymorphone available in USA
1960	Fentanyl synthesized
1964	World Health Organization introduces concept of opioid dependence
1965	USA estimates that 750,000 citizens are addicted to heroin
1967	Talwin (pentazocine) approved for pain relief and is described as having no known potential for abuse
1968	First reports of Talwin dependence
1968	Bureau of Narcotics and Dangerous Drugs established in the Justice Department
1970	Congress passes Controlled Substances Act
1973	Drug Enforcement Administration (DEA) is set up under the Justice Department
1979	Schedule IV controlled substance act, labeling changes to include postmarketing events of addiction
1982	Talwin is reformulated to include naloxone and marketed commercially the following year
1983	The original formulation of Talwin (without naloxone) is withdrawn from market and reports of abuse decreased in next few years
1999	Veterans Health Administration launches the “Pain as the 5th Vital Sign” initiative. JCAHO and other regulatory bodies incorporate into their guidelines, which was initial start of increased opioid prescriptions
2000	Congress declares decade 2001–2010 “Decade of Pain Control and Research”
2002	Suboxone (buprenorphine/naloxone) approved
2004	Consumer lawsuit against Purdue Pharma regarding OxyContin
2004	First “around-the-clock” product approved for opioid-tolerant pain patients (Palladone, Purdue Pharma)
2005	Palladone pulled from the USA market (still available in UK)
2005	Majority of single-agent oxycodone sold in US is extended release (64%)
2007	Reports of Suboxone abuse nationally as abusers figured out how to extract buprenorphine
2007	USA consumes 82% of world's supply of oxycodone annually
2009	Embeda (morphine with sequestered naltrexone) approved
2009	Majority of single-agent oxycodone sold in US is immediate release (54%)
2010	Safe use Initiative launched by FDA

**Table 2:** Definitions.

**Aberrant drug-related behaviors:** behaviors that depart or deviate from strict adherence to the prescribed therapeutic regimen set forth by a physician. Some examples include [9, 20] (list is not exhaustive)	
(i) Forging prescriptions	
(ii) Stealing or borrowing drugs	
(iii) Multiple episodes of loss or theft of prescription drugs	
(iv) Not following prescribed dose and schedule on several occasions	
(v) Using prescribed drugs before expected renewal date	
(vi) Injecting or snorting opioids	
(vii) Multiple unauthorized dose increases (self-escalating)	
(viii) Obtaining drugs from friends, family, street, and others	
(ix) Repeatedly seeking drugs from other providers or emergency rooms	
(x) Concurrent use of illicit drugs (e.g., heroin, cocaine, methamphetamine, marijuana, and others)	
(xi) Concurrent use of alcohol	
(xii) Past history of abuse of prescription medications, and possibly street drugs	
(xiii) Requests for specific drugs, especially a preference for immediate release over sustained release preparations	
(xiv) Increase in anxiety, sleep disturbance, or depression	
(xv) Urine drug test positive for illicit drugs or unauthorized drugs	
(xvi) Doctor shopping	
(xvii) Persistent oversedation or euphoria	
(xviii) Appearing intoxicated	
(xix) Deterioration of function at work, in the family, or socially	
(xx) Decrease in physical, psychological, or social function	
(xxi) Noncompliance with nonopioid components of pain treatment	
(xxii) Reporting no effect from nonopioids, especially antidepressants	
(xxiii) Noncompliance with nondrug components of pain treatment (psychotherapy, PT, etc.)	
(xxiv) Accidents: motor vehicle, falls, and others	

**Addiction**: addiction is a primary, chronic disease of brain reward, motivation, memory, and related circuitry. Dysfunction in these circuits leads to characteristic biological, psychological, social, and spiritual manifestations [21].	

**Substance abuse**: a maladaptive pattern of substance use leading to clinically significant impairment or distress as manifested by one (or more) of the following, occurring within a 12-month period [22].	
(1) Recurrent substance use resulting in a failure to fulfill major role obligations at work, school, or home (such as repeated absences or poor work performance related to substance use; substance-related absences, suspensions, or expulsions from school; or neglect of children or household).	
(2) Recurrent substance use in situations in which it is physically hazardous (such as driving an automobile or operating a machine when impaired by substance use).	
(3) Recurrent substance-related legal problems (such as arrests for substance-related disorderly conduct).	
(4) Continued substance use despite having persistent or recurrent social or interpersonal problems caused or exacerbated by the effects of the substance (e.g., arguments with spouse about consequences of intoxication and physical fights).	

**Dependence**: physical dependence isa state of adaptation that is manifested by a drug class-specific withdrawal syndrome that can be produced by abrupt cessation, rapid dose reduction, decreasing blood level of the drug, and/or administration of an antagonist [23].	

**Table 3:** Opioid analgesic formulations designed to resist or deter abuse which are commercially available or in development.

Name	Company	Agent(s)	Description	Comment
Physical barrier				

Remoxy [24]	Pain therapeutics and King Pharmaceuticals	Oxycodone extended release	Hard gelatin capsule containing viscous liquid	ORADUR technology (extended-release formulation)
Acuracet [25]	Acura	Oxycodone immediate release and acetaminophen	Impediments to snorting and injection (not further described)	
Vycavert [26]		Hydrocodone immediate release and acetaminophen	Impediments to snorting and injection (not further described)	
C0L-003 [27]	Collegium pharmaceutical	Oxycodone sustained release	Multiparticulate matrix with particles in waxy excipient base	
COL-172 [27]		Oxycodone sustained release	Multiparticulate matrix with particles in waxy excipient base	
ReXista [28]	Intellipharmaceutics	Oxycodone sustained release	Not described	
OxyContin [29]	Purdue	Oxycodone controlled release	Resists crushing and dissolving	
TQ-1015 [30]		Oxycodone extended release	Crush and tamper resistant	Securel technology
TQ-1017 [31]	TheraQuest Biosciences	Tramadol extended release	Transforms into viscous substance when hydrated	
TQ1020 [32]		Levorphanol extended release	Not stated	
DDS-08B [33]	Labopharm/Paladin	Oxycodone/APAP extended release	Extended release even if tablet is tampered with	
Exalgo [34]	Neuromed/Covidien	Hydromorphone extended release	Osmotic delivery system (OROS push-pull technology)	New formulation
Not named [35]	Pisgah Labs	Hydrocodone	Insoluble at pH ranges in mucosal membranes	
ADPREM [36, 37]	Egalet (Denmark)	Morphine	Erodible matrix covered by water-impermeable nonerodible shell	
Egalet hydrocodone [36]	Egalet (Denmark)	Hydrocone	Hard matrix	

Agonist/Antagonist				

Embeda [38]	King Pharmaceuticals	Morphine/naltrexone	Pellets of morphine surrounding a hard core of sequestered naltrexone	Naltrexone pellets are 1.0 to 1.7 mm diameter
ALO-01 [39]	Alpharma	Morphine extended release with naltrexone	Sequestered naltrexone	
Oxytrex [40]	Albert Einstein College of Medicine	Oxycodone and ultra-low-dose naltrexone	Ultra-low-dose naltrexone	
OxyNal or ELI-216 [40]	Elite Pharmaceuticals	Oxycodone-controlled release with naltrexone pellets	Pellets release naltrexone only when crushed	
Talwin [41]	Sanofi-Aventis	Pentazocine and naloxone	Naloxone	Naloxone released if drug administered parenterally
Suboxone Film [42]	Reckitt Benckiser	Buprenorphine/naloxone	Naloxone	

Aversive agents				

Acurox with niacin [43]	Acura Pharmaceuticals and King Pharmaceuticals	Oxycodone immediate release 5 and 7.5 mg	Niacin; tablets contain a gel-forming ingredient	

Prodrug				

NRP 290 [44, 45]	New River Pharmaceuticals	Conditional bioreversible derivative of hydrocodone	Lysine-modified prodrug	NRP 369 is backup for NRP 290
